# Association between mesothelioma and non-occupational asbestos exposure: systematic review and meta-analysis

**DOI:** 10.1186/s12940-018-0431-9

**Published:** 2018-12-19

**Authors:** Rengyi Xu, Frances K. Barg, Edward A. Emmett, Douglas J. Wiebe, Wei-Ting Hwang

**Affiliations:** 1 Department of Biostatistics, Epidemiology, and Informatics, Perelman School of Medicine, University of Pennsylvania, Philadelphia, PA 19104 USA; 2 Department of Family Medicine and Community Health, Perelman School of Medicine, University of Pennsylvania, Philadelphia, PA 19104 USA; 3 Department of Environmental and Occupational Medicine, Perelman School of Medicine, University of Pennsylvania, Philadelphia, PA 19104 USA; 4 Penn Superfund Research Program, Perelman School of Medicine, University of Pennsylvania, Philadelphia, PA 19104 USA; 5 Department of Biostatistics, Epidemiology, and Informatics, Perelman School of Medicine, University of Pennsylvania, 902 Blockley Hall, 423 Guardian Drive, Philadelphia, PA 19104-6021 USA; 6 Department of Biostatistics, Epidemiology, and Informatics, Perelman School of Medicine, University of Pennsylvania, 610 Blockley Hall, 423 Guardian Drive, Philadelphia, PA 19104-6021 USA

**Keywords:** Non-occupational exposure pathways, Asbestos, Mesothelioma, Systematic review, Meta-analysis

## Abstract

**Background:**

The risk of mesothelioma has been shown to be associated with exposure to asbestos fibers. Most of the existing literature focuses on occupational exposure; however, non-occupational asbestos exposure has also been identified as an important risk factor.

**Objective:**

To estimate the association between mesothelioma and non-occupational asbestos exposure, and evaluate control recruitment and exposure measurement methods.

**Methods:**

A systematic literature review was conducted to identify case-control (CC) and cohort studies that examined the association between mesothelioma and non-occupational exposure to asbestos, including neighborhood, domestic, and household exposure. Meta-analysis was performed to estimate a summary relative risk estimate (SRRE) and 95% confidence interval using random-effects models. Subgroup analyses were also conducted by exposure type, gender, region, and fiber type.

**Results:**

Twenty CC and 7 cohort studies were selected. Controls in CC studies were selected from the general population (55%), hospital records (18%), cancer registry (23%) and a combination of population and hospital records (5%). Multiple methods were used to measure neighborhood exposure (e.g., linear distance and direction of residence from an asbestos factory), domestic (e.g., whether living with an asbestos worker) and household exposure (e.g., whether involved in asbestos-containing home improvement projects). Primary meta-analyses suggested a SRRE of mesothelioma of 5.33 (95%CI: 2.53, 11.23) from neighborhood exposure, 4.31 (95%CI, 2.58, 7.20) from domestic exposure, and 2.41 (95%CI, 1.30, 4.48) from household exposure with large I^2^ statistics ranging from 83**–**99%.

**Conclusions:**

Non-occupational asbestos exposure is significantly associated with an elevated risk of mesothelioma. Funnel plots indicated a potential of publication bias. Some SRREs should be interpreted with cautions because of high between-studies heterogeneity.

## Background

The relationship between mesothelioma and exposure to asbestos has been the focus of a large body of research spanning decades. Mesothelioma is a rare cancer with very poor prognosis. Five-year survival rates are less than 5%, and the median survival for malignant mesothelioma patients is only 1 year [1, 2]. Asbestos became widely used as an important industrial resource in many countries from the late 19th and early 20th centuries. It was used in the United States into the 1970s and is still used in some developing countries.

Between 1999 and 2015, the annual number of deaths from malignant mesothelioma increased from 2479 to 2597 in the United States. The rate of death, alternatively, was estimated to have decreased over that period from 13.96 to 10.93 deaths per 1 million persons. These rates are a challenge to project, and they did not specifically consider cases of mesothelioma resulting from non-occupational exposure [2].

Exposure to asbestos in an occupational context – including mining asbestos or working in a factory that uses asbestos in the manufacturing process – is a critical risk factor that is associated with an increased likelihood for developing mesothelioma [3, 4]. Some review studies have also shown that non-occupational exposure to asbestos also results in an elevated risk of mesothelioma, [5–9] however considerably less research has been dedicated to this topic and with less consistent results. This is likely due in part to the challenges of measuring and classifying exposure that occurs outside the occupational setting.

Definitions of different types of non-occupational exposure to asbestos are varied in the literature with several similar and sometimes confusing exposure pathway nomenclature [10]. Non-occupational asbestos exposures are generally divided into three sources according to the exposure pathway: neighborhood, household, and domestic. Neighborhood exposure generally refers to exposure results from living near asbestos factories or sites of naturally occurring asbestos (NOA). Household exposure results from having exposure to asbestos-containing materials used in home structures (e.g., roofs, insulation), from home-based hobby (e.g., gardening), or home improvement projects. Domestic exposure refers to exposure to fibers brought home by asbestos workers on their clothing or in their hairs or through living in the same house with occupationally exposed individuals. Other terms also used to describe non-occupational exposure in the literature such as residential exposure, which is related to neighborhood exposure, and the term environmental exposure which often includes neighborhood and household exposure but not domestic exposure. On the other hand, the term paraoccupational- or household-contact exposure is often used interchangeably with domestic exposure.

We conducted a systematic review and meta-analysis of studies that have investigated the association of mesothelioma and different non-occupational sources of exposure using either a case-control or cohort study design. Our goal was to use this literature to estimate the relative risk of the development of mesothelioma among subjects who were exposed to asbestos through non-occupational pathways. We also evaluated the methods used for control recruitment and for exposure measurement in the selected studies, and discuss advantages and disadvantages of different approaches.

## Methods

We followed guidelines from Preferred Reporting Items for Systematic Reviews and Meta-Analyses (PRISMA) statement [11] and Meta-analysis Of Observational Studies in Epidemiology (MOOSE) guideline [12] to conduct and report our study results.

### Search strategy

A comprehensive literature search was conducted in PubMed to identify articles on association between mesothelioma and non-occupational asbestos exposure. There was no restriction on the publication date, and the keywords we used were ‘mesothelioma AND asbestos AND (cohort OR case-control OR case control)’. Additional records were also identified through sources other than the PubMed database, including the reference list from the PubMed identified articles.

### Inclusion and exclusion criteria

We excluded studies that were review articles, meta-analysis articles, and those that did not focus on the association of interest. The eligibility criteria to be included in the analysis were: 1) case-control or cohort design; 2) analysis of the association between mesothelioma and non-occupational asbestos exposure; studies that focused on only occupational associations were not included. In addition to these criteria, studies also must have either reported an effect estimate and 95% confidence interval (CI), or included sufficient information for us to calculate a crude measure of association and variance. Measures of association included relative risk (RR), odds ratio (OR), and standardized incidence ratio (SIR).

### Data extraction

The following information was extracted from each study: author, publication year, study design, data source (hospital records, cancer registry, local health authority, etc.), country of study site (UK, Italy, Canada, United States, etc.) and region (Europe, Africa, etc.), inclusion year of subject enrollment, sample size, definition of case and control, source of case and control, disease type (pleura or peritoneal mesothelioma, mixed, etc.), fiber type (e.g., crocidolite, chrysotile, mixed, etc.), exposure type (neighborhood, household, domestic, mixed), measurement of exposure, gender (male only, female only, mixed), statistical analysis method (regression, test, adjusted, unadjusted), effect estimate, and 95% CI.

### Study quality scoring

Selected studies were assigned a score based on the quality of their study designs. We adapted the quality scoring methodology that used by Garabrant et al. [13] to study asbestos-mesothelioma association. Our scoring methodology included the following ten criteria: 1) Specific definition of asbestos exposure types provided? (no = 0; yes = 1); 2) Lifetime asbestos exposure history obtained? (no = 0; yes = 1); 3) Potential confounding issues addressed? (no = 0; yes = 1); 4) Exposure-response analysis performed? (no = 0; yes = 1); 5) For cohort studies, duration of follow-up greater than 30 years? (no = 0; yes = 1); 6) For case-control studies, participation rate greater than 80%? (no = 0; yes = 1); 7) Information bias on outcome (disease)? possible = 0 (e.g., self-reported outcome); unlikely/addressed = 1 (e.g. records obtained from cancer registry or hospital records); 8) Information bias on exposure measurement? possible = 0 (e.g. self-reported measures); unlikely/addressed = 1 (e.g. measures from official records); 9) Selection bias? possible = 0 (e.g. in hospital-based case-control studies); unlikely/addressed = 1 (e.g. in cohort studies or population-based case-control studies); 10) Cases confirmed by pathologic review? (no = 0; yes = 1). Based on the total score, we divided the studies into three tiers according to the total quality score (tier 1: score 8 to 10, tier 2: score 6 to 7, and tier 3: score 0 to 5).

### Summary measures

Studies reported different measures of association, including RR, OR, and SIR. In the context of our study, OR ≈ RR because mesothelioma is a rare disease, and RR ≈ SIR since the exposure rate is relatively low in the whole population. Therefore, the effect estimate of OR and SIR are approximately equal to the effect estimate of RR [14]. We then combined the different measures of association and calculated a summary relative risk estimate (SRRE) and the associated 95% CI.

### Statistical analyses

To combine estimates across different studies, we used random-effects models on the log of the risk estimates (e.g., RR) with DerSimonian and Laird [15] estimation in the meta-analysis. In a random-effects model, the true risk varies across studies; each follows a distribution with its own mean and variance. The statistical weights used to pool the study-specific estimates is based on the inverse of the total variance which sum over the between and within variation across the studies. We used a random-effects model because the assumption under a fixed effects model for a common effect size among the selected studies would be unreasonable.

To distinguish the effects on mesothelioma from different non-occupational exposures, we analyzed the data by asbestos exposure types: neighborhood, domestic, and household. Mixed exposure types are also possible. Because the determination of the exposure types can be difficult without the original data, we classified the exposure type based on study authors’ designation except for two studies, [16, 17] where sufficient descriptions were available for us to re-assign exposure types (household exposure to domestic exposure or vice versa). If a study did not specifically use the terms of neighborhood, domestic, and household, we assigned the exposure types according to the texts provided by the authors based on the definitions described in the introduction section. Three studies [18–20] reported gender-specific association and did not provide a point estimate on the association for the entire study population, but we were able to hand calculate a crude OR and its 95%CI for both males and females using the information provided in the article. Data for one cohort study were reported in two publications, [16, 21] and only the updated estimates from the more recent publication were used. Results of the meta-analysis were reported using forest plots. We also reported the I^2^ statistic, which indicates the percentage of variation attributable to study heterogeneity (25% low heterogeneity, 50% medium, 75% high), to quantify inconsistency [22]. Funnel plots, which plot point estimates against study precision, were generated to investigate publication bias. We used standard error in our analysis as the measure for study precision. When there is no publication bias or systematic heterogeneity among the studies, we should expect a symmetric inverted funnel shape. Subgroup analysis was conducted to further assess the association of interest by gender, fiber type, study region, and study design. We also compared the results within the three tiers of quality scoring. The statistical analysis was conducted using the *metafor* package [23] in R version 3.4.0 (R foundation, Vienna, Austria).

## Results

**Fig. 1:**
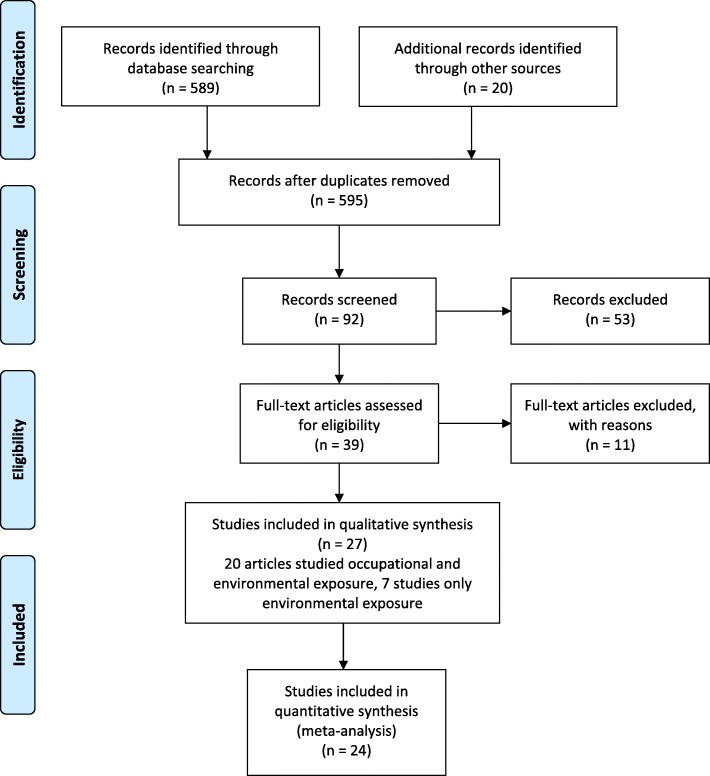
PRISMA flow diagram of included studies

Five hundred eighty-nine articles were found through PubMed search, as well as 20 that were identified by additional sources (e.g., reference of a selected article). After further screening, 27 studies were found to be eligible and were included in the qualitative synthesis of the systematic review, and 24 studies were included in the final meta-analysis cohort (Fig. 1). Among the eligible studies, 20 (74%) were case-control, and 7 (26%) were cohort studies.

### Exposure measurement

As shown in Table 1, 14 studies [16, 19, 24–35] reported a risk effect estimate for mesothelioma that attributed specifically to neighborhood exposure, 12 studies [16–18, 24, 33–40] reported estimates for domestic exposure, and 5 studies [16, 17, 26, 33, 41] examined household exposure. Several studies reported separate risk estimates for multiple exposure types. One study [42] did not distinguish neighborhood and domestic exposures and reported a combined effect point estimate, and one study [31] reported a combined effect estimate for domestic and household exposure. Additionally, one study [20] reported one effect estimate as non-occupational exposure and did not distinguish different exposure types.

**Table 1:** Summary of studies and corresponding relative risk estimates (95% CI) included in the meta-analysis

Author (Year)	Country	Design	Inclusion year	Fiber	Case source	Control source	Sample size	Effect estimate (95% CI)	Quality Tier^c^
Neighborhood									
Mensi (2015)	Italy	CO	2000–2011	Ch, Cr	Ca. registry	NA	3455	6.6 (5.2, 8.3)	2
Bayram (2013)	Turkey	CC	2000–2010	NR	Ca. registry	Registry	532	1.88 (1.63, 2.16)	2
Tarres (2013)	Spain	CO	2000–2009	NR	Hospital	NA	24	12.92 (10.77, 15.33)	2
Madkour (2009)	Egypt	CC	2003–2004	Ch	Central agency	Pop	4291	27.89 (3.89, 200.1)	2
Musti (2009)	Italy	CC	1993–2003	Mixed	Ca. registry	Pop	273	5.29 (1.18, 23.74)	1
Baumann (2007)	New Caledonia	CC	1984–2002	NR	Ca. registry	Registry	188	2.49 (0.82, 7.58)	3
Maule (2007)^a^	Italy	CC	1987–1993	Mixed	LHA	Pop	272	5.1 (1.9, 13.4)	1
Pan (2005)	USA	CC	1988–1997	NR	Ca. registry	Registry	2908	0.93 (0.90, 0.98)	1
Metintas (2002)	Turkey	CO	1990–2000	Tr, Ch	Ca. registry, hosp, LHA	NA	1886	77.17 (51.73, 115.1) ^d^	3
Magnani (2000)	Multi-countries^b^	CC	1995–1996	NR	Ca. registry	Pop, hosp	448	11.5 (2.84, 46.5)	1
Rees (1999)	South Africa	CC	1988–1990	Am,Cr	Hosp	Hosp	345	19.6 (3.7, 105)	2
Howel (1997)	UK	CC	1979–1991	Mixed	Ca. registry	Registry	345	6.6 (0.86, 59)	2
McDonald (1980)	Canada, USA	CC	1960–1972	Ch	Pathologic societies	Hosp	490	0.25 (0.03, 2.24)	3
Newhouse(1965)	UK	CC	1989–2001	Mixed	Hosp	Hosp	152	5.46 (1.72, 17.31)	3
Domestic									
Ferrante (2016)	Italy	CC	2001–2006	NR	LHA	Pop	348	2.4 (1.3, 4.4)	2
Mensi (2015)	Italy	CO	2000–2011	Ch, Cr	Ca. registry	NA	3455	8.7 (6.2, 12.1)	2
Rake (2009)	UK	CC	2001–2006	NR	Ca. registry, hosp	Pop	1420	2 (1.3, 3.2)	2
Ferrante (2007)	Italy	CO	1907–1986	NR	Registrar’s office	NA	1780	25.19 (12.57, 45.07)	1
Maule (2007)^a^	Italy	CC	1987–1993	Mixed	LHA	Pop	272	1.4 (0.7, 2.9)	1
Welch (2005)	USA	CC	1989–2001	NR	Ca. registry	Registry	48	5.5 (1.03, 29.45)	3
Case (2002)	Canada	CC	1970–1989	Ch	Hosp	Pop	160	4.92 (0.61, 39.87)	2
Howel (1997)	UK	CC	1979–1991	Mixed	Ca. registry	Registry	345	5.8 (1.7, 19.2)	2
Spirtas (1994)	USA	CC	1975–1980	NR	Ca. registry, hosp	Pop	533	1.77 (0.92, 3.4) ^d^	2
McDonald (1980)	Canada, USA	CC	1960–1972	Ch	Pathologic societies	Hosp	490	4.0 (0.85, 18.84)	3
Vianna (1978)	USA	CC	1967–1977	NR	Registrar’s office	Pop	52	10 (1.42, 37.4)	3
Newhouse(1965)	UK	CC	1989–2001	Mixed	Hosp	Hosp	152	16.75 (2.04, 137.51)	3
Household									
Ferrante (2016)	Italy	CC	2001–2006	NR	LHA	Pop	348	2.0 (1.2, 3.2)	2
Baumann (2007)	New Caledonia	CC	1984–2002	NR	Ca. registry	Registry	188	1.01 (0.98, 1.04)	3
Ferrante (2007)	Italy	CO	1907–1986	NR	Registrar’s office	NA	1780	2 (1.2, 3.2)	1
Maule (2007)^a^	Italy	CC	1987–1993	Mixed	LHA	Pop	272	1.3 (0.8, 2.3)	1
Luce (2000)	New Caledonia	CC	1993–1995	Tr	Ca. registry	Pop	305	40.9 (5.15, 325)	2
Neighborhood/Domestic									
Reid (2008)	Australia	CO	1950–2004	Ch, Cr	Ca. registry, hosp	NA	2552	2.08 (0.8, 5.42)	1
Domestic/Household									
Magnani (2000)	Multi-countries^b^	CC	1995–1996	NR	Ca. registry	Pop	448	4.92 (1.78, 13.6)	1
Neighborhood/Domestic/Household									
Lacourt (2014)	France	CC	1998–2002	NR	PNSM	Pop	874	3.84 (1.27, 11.58) ^d^	1

*Abbreviations*: *Ca. registry* Cancer registry, *CC* Case-control, *CO* Cohort, *Ch* Chrysotile, *Cr* Crocidolite, *Tr* Tremolite, *Am* Amosite, *NR* Not Reported, *M* Mesothelioma, *Pop* Population-based, *Hosp* Hospital-based, *LHA* Local health authority, *PNSM* National program for mesothelioma surveillance, *NA* not applicable

^a^: updated results for Magnani (2001) ^b^:Includes Italy, Spain and Switzerland. ^c^: Quality tiers: Tier 1 (Quality score: 8–9), Tier 2 (Quality score: 6–7), Tier 3 (Quality score; 4–5).^d^:calculated from the gender-specific estimates

The selected studies used different methods to quantify the different types of exposure. For neighborhood exposure, the most popular method, used by 12 studies [25, 26, 28–30, 33–35, 37, 40–42] was linear distance between residence and nearest asbestos site. Five studies [16, 20, 21, 31, 36] assigned a score based on probability, intensity and duration of neighborhood exposure to each subject, and three studies [27, 32, 42] measured the duration of time spent in an asbestos exposed district. Outdoor airborne asbestos fiber concentration was also used as a measure by two studies [19, 27].

For domestic related exposure, eight studies [18, 24, 34, 35, 37–40] used a binary indicator of whether the subject had lived with a family member exposed to asbestos at work, and one study [17] further took the duration of the working history of subject’s family member into consideration. Howel et al. [33] divided the subjects into three groups based on the likelihood of having a domestic exposure. Lacourt et al. [20] assigned a score based on probability, intensity and frequency of exposure. Ashcroft et al. [43] estimated the fiber counts in the subject’s lung tissue.

For household related exposure, four studies [16, 21, 37, 41] considered a binary indicator of whether the subject had been involved in any activities that might include asbestos-containing products, such as whitewash (e.g., “po”) or stucco for walls and roofs of houses. Three studies [20, 31, 36] assigned a score based on probability, frequency and intensity of exposure from do-it-yourself project activities that might involve asbestos-containing products.

Andersen et al. [44] investigated a different pathway of exposure, where the asbestos exposure resulted from drinking water from wells that received rain water off asbestos-cemented-tiled roofs.

### Control recruitment

In the 20 selected case-control studies, controls were selected in three ways: population-based, hospital-based and combined population- and hospital-based. Twelve studies [16, 18, 20, 21, 27, 28, 31, 36, 37, 39–41] selected controls from among general population, four studies [32, 34, 35, 43] recruited controls from the same hospital that were used to select cases, five studies [25, 29, 30, 33, 38] used the same cancer registry from which cases were selected, and one study [31] selected controls from both the general population and hospitals. Sources of data for the studies that used population-based controls included state health department, local health authority, electoral rolls, and births and deaths registry. Studies that used hospital-based controls selected patients with a diagnosis other than mesothelioma. For example, Rees et al. [32] excluded patients with lung, pleural or peritoneal diseases and also those with conditions of central nervous system. Newhouse et al. [35] selected patients from medical and surgical wards of the hospital, which did not include subjects with mesothelioma or mesothelioma related diseases.

Five case-control studies selected controls from a cancer registry, which indicates all the subjects were diagnosed with some type of cancer that is not related to asbestos exposure. The authors either included patients with certain cancer types or excluded patients with certain cancer types. For example, Welch et al. [38] selected patients with appendiceal cancer as controls, and Pan et al. [30] only included malignant cancer pancreatic patients. Bayram et al. [25] selected prostate or breast cancer patients as controls. On the other hand, Howel et al. [33] excluded any patient who died from mesothelioma, or diseases that could have been confused with mesothelioma, and Baumann et al. [29] excluded patients with the pleura, peritoneal or lung cancer.

One case-control study, by Magnani et al., [31] recruited controls from both general population and hospitals and collected data from multicenter studies; they used population-based controls for two centers (Italy and Switzerland), and hospital-based controls for one center (Spain).

To accomplish a reasonable comparison between the cases and controls, all studies used certain factors to match the controls with cases. The most commonly used factors to match controls to cases were gender and 5-year age groups. Some studies further matched by time of diagnosis, [29, 40] place of residence, [18, 20, 40] birthplace, [25] vital status, [16, 21] marital status, [40] and year of death [16, 21, 28, 33].

### Primary meta-analysis results

**Fig. 2:**
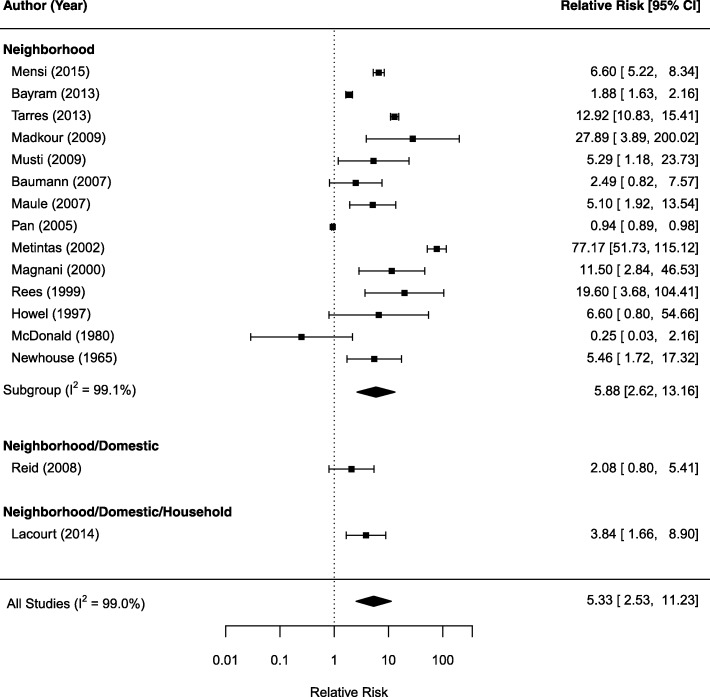
Forest plot of relative risk estimates (95% CI) for mesothelioma as related to neighborhood exposure

**Fig. 3:**
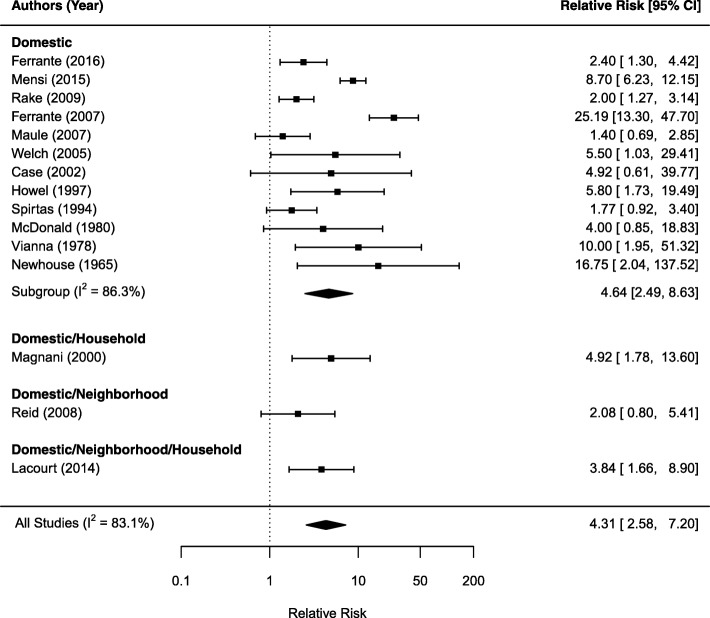
Forest plot of relative risk estimates (95% CI) for mesothelioma as related to domestic exposure

**Fig. 4:**
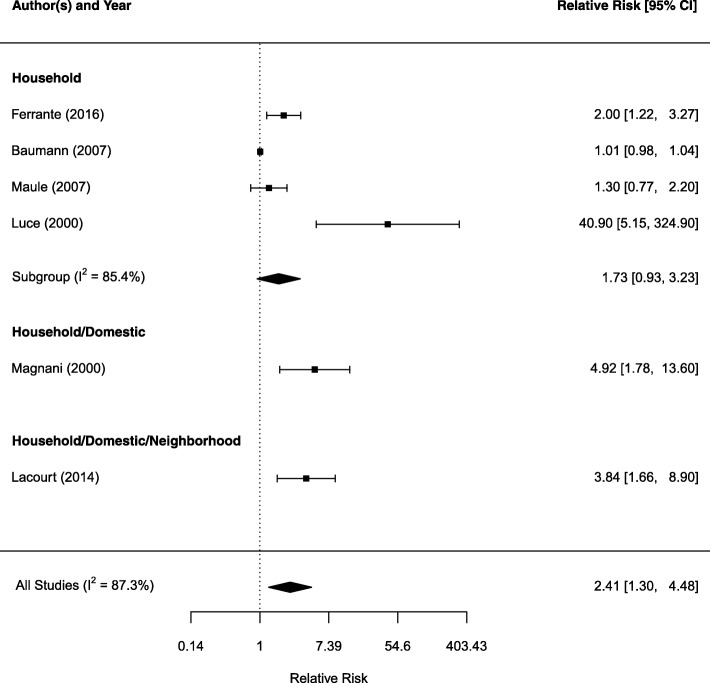
Forest plot of relative risk estimates (95% CI) for mesothelioma as related to household exposure

We examined the relative risk of mesothelioma by exposure type. Figures 2, 3, and 4 show the forest plot for neighborhood (Fig. 2), domestic (Fig. 3), and household exposure (Fig. 4), respectively. In Fig. 2, 16 studies reported relative risk estimates for either neighborhood only (14 studies) or mixed neighborhood/domestic exposure (one study) or neighborhood/domestic/household (one study). The SRRE from the random-effects model is 5.33 (95%CI: 2.53, 11.23). The I^2^ statistic was 99%, indicating considerable heterogeneity among the studies. This high heterogeneity was mainly driven by the result from Mentinas et al., [19] which reported a relative risk as high as 77.17. Removing this study resulted in a SRRE of 4.31 (95%CI: 2.16, 8.61). Among 14 studies reporting a relative risk associated with neighborhood only exposure, the SRRE is 5.88 (95% CI: 2.62, 13.16).

Figure 3 shows the forest plot on domestic exposure. Twelve studies examined domestic exposure only gives an estimated SRRE of 4.64 (95%CI: 2.49, 6.63) with I^2^ statistic of 86.3%, indicating a large heterogeneity among the studies. Combing three other studies with a mixed neighborhood and/or household exposure resulted in a SRRE of 4.31 (95%CI: 2.58, 7.20). Figure 4 shows the forest plot that examined household and mixed household exposures with neighborhood or domestic exposures. Among four studies examined household exposure only shows a non-significant SRRE of 1.73 (95%CI: 0.93, 3.23) with I^2^ statistic of 87.3% for high between study heterogeneity. Considering all six studies with any household exposure produces a SRRE of 2.41 (95%CI: 1.30, 4.48) and I^2^ statistic of 87.3%. Removing the study by Luce et al., [41] which reported the largest RR of 40.90 in this subgroup, reduced the SRRE to 1.92 (95%CI: 1.30, 4.48).

### Subgroup analysis results

We examined the effect of asbestos exposure on mesothelioma by gender, fiber type, study region, and study design. Table 2 reports the SRREs (95% CI) and number of selected studies by exposure type (overall, any neighborhood, any domestic and any household exposure). The effect of asbestos exposure for gender varied across different exposure types. The SRRE of any non-occupational exposure was 4.80 (95%CI: 1.96, 11.76) for males and 7.83 (95% CI: 3.30, 18.57) for females. Similar magnitudes of SRREs are obtained for neighborhood and domestic exposures for males and females ranging from SRRE of 3.24 for domestic exposure for males to SRRE of 7.69 for neighborhood exposure for females. Considering any non-occupational exposures, three studies reported chrysotile-specific RR combined into a non-significant SRRE of 3.56 (95%CI: 0.65 to 20.83). Majority of the studies either reported a mixed fiber types (6 studies, SRRE of 7.17, 95%CI: 3.36, 15.28) or did not report the fiber types (11 studies, SRRE of 2.66, 95%CI: 1.89, 3.74). More than half of the studies (13 studies) were done in Europe, the SRRE is 5.44 (95%CI: 3.16, 9.37) compared to 2.15 (95%CI: 1.01, 4.56) for USA/Canada (6 studies), 22.27 (95%CI: 6.35, 81.32) for South Africa/Egypt (2 studies), and 2.64 (95%CI: 0.92, 7.57) for Australia/South Pacific Island of New Caledonia (3 studies). Combined risk estimates from a small number of cohort studies had ranged from 8.17 to 12.62 depending different types of exposure; they are also higher than the SRREs from case-control studies that are in the range of 2 to 4. Between-study heterogeneity is large for all the subgroup meta-analyses with a median of I^2^ statistics of 87.5%. Because only half of the selected studies reported the exclusive use of pleural mesothelioma patients while the other half of the studies did not report enough information to discern whether pleural, peritoneal or mixed types of mesothelioma were analyzed, thus no subgroup analysis on mesothelioma types was performed.

**Table 2:** Summary relative risk estimates (SRRE), 95% confidence intervals (CI)†, and number of studies in subgroup analyses

	SRRE (95% CI)	No. Studies	SRRE (95% CI)	No. Studies	SRRE (95% CI)	No. Studies	SRRE (95% CI)	No. Studies
	Overall Exposure		Neighborhood		Domestic		Household	
Gender								
Male	4.80 (1.96, 11.76)	7	5.71 (1.18, 27.54)	4	3.24 (1.63, 6.45)	5	2.40 (0.2, 26.70)	1
Female	7.83 (3.30, 18.57)	10	7.69 (1.91, 31.00)	5	6.13 (2.42, 15.51)	8	4.30 (1.20, 15.1)	1
Fiber type								
Chrysotile	3.56 (0.65,20.82)	3	2.70 (0.03, 273.75)	2	4.30 (1.24, 14.94)	2	–	0
Mixed	7.63 (3.68, 15.82)	8	10.36 (3.24, 33.16)	7	7.40 (2.69, 20.37)	5	1.30 (0.80,2.30)	1
Not reported	2.66 (1.89, 3.74)	11	3.53 (1.27, 9.81)	6	2.70 (1.90, 3.85)	7	2.23 (1.06, 4.70)	4
Region								
Europe	5.44 (3.16, 9.37)	13	7.59 (3.24, 17.77)	10	4.82 (2.46, 9.44)	9	2.37 (1.34,4.19)	4
USA/Canada	2.15 (1.01, 4.56)	6	0.77 (0.30, 1.95)	2	3.36 (1.68, 6.72)	5	–	0
South Africa/Egypt	22.27 (6.35, 81.32)	2	22.27 (6.35, 81.32)	2	–	0	–	0
Australia/South Pacific	2.64 (0.92, 7.57)	3	2.55 (1.09, 4.64)	2	2.08 (0.80, 5.42)	1	5.53 (0.15,205.3)	2
Study Type								
Cohort	12.62 (6.30, 25.26)	5	11.59 (4.37, 30.79)	4	8.17 (2.77, 24.13)	3	–	0
Case-Control	2.12 (1.76, 2.56)	19	3.37 (2.08, 5.46)	12	2.88 (2.05, 4.03)	12	2.41 (1.30, 4.48)	6

†: *p*-value< 0.05 for statistically significant SRRE if 95%CI does not include 1

**Fig. 5:**
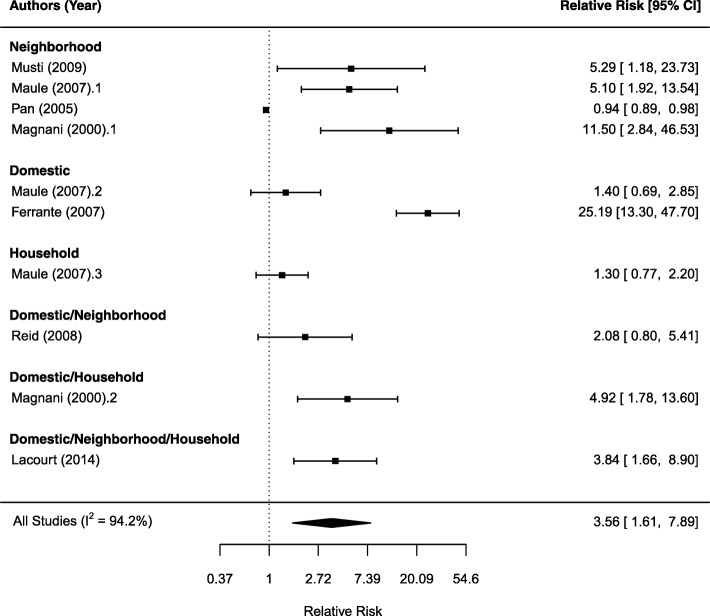
Forest plot of relative risk estimates (95% CI) for mesothelioma as related to non-occupational exposure using studies with tier 1 quality score

We also performed subgroup analyses on the studies by tier of quality scores. In Figs. 5, 7 high quality (Tier 1) studies combine to a SRRE of 3.56 (95%CI: 1.61, 7.89). The I^2^ statistic was 94.2%, which shows heterogeneity across individual studies. Studies in Tier 2 reported a SRRE of 4.94 (95%CI: 2.89, 8.22), with I^2^ statistic of 94.9%. Studies in Tier 3 reported a SRRE of 4.88 (95%CI: 1.63, 14.59), with I^2^ statistic of 95.4%. All tiers reported a significant association between asbestos exposure and the risk of mesothelioma regardless a large heterogeneity.

### Publication bias

**Fig. 6:**
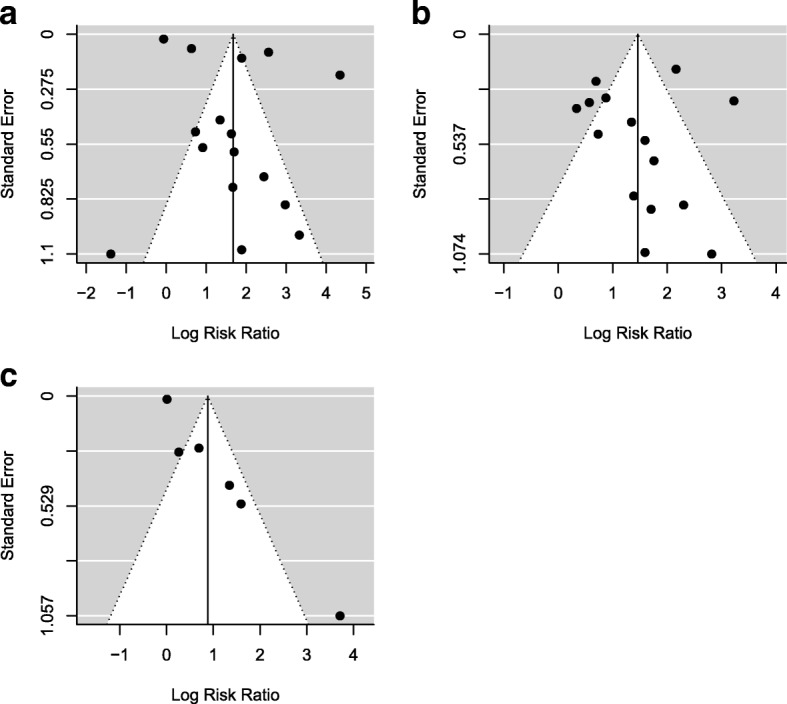
Funnel plots among studies on (**a**) neighborhood exposure; (**b**) domestic exposure; (**c**) household exposure

We generated funnel plots to investigate publication bias by exposure types among the selected studies. As shown in Fig. 6, studies in all three exposure types indicated potential existence of publication bias in which most studies published have a positive relative risk estimate and small standard error (right-hand side in the triangle area) while lack of studies that showed negative association (left-hand side).

## Discussion

This report describes a systematic review of exposure assessment, control selection methods, and a series of meta-analyses to estimate overall effects of non-occupational asbestos exposure and the risk of mesothelioma associated with different types of exposure (neighborhood, household, domestic). We found that several methods have been used to operationally assess asbestos exposure, with varying levels of complexity ranging from a simple distance measure to a score that based on both probability and intensity of exposure. There were no clear advantages or disadvantages to a simple or complicated approach other than practical and logistical considerations. In general, exposure assessment will not be accurate if definitions for exposure types or pathway are not clearly defined. More broadly for the field, efforts to understand how different exposure types relate to risk of mesothelioma will be hampered if exposure definitions are not reported clearly or are interpreted differently by researchers. In particular, we found that differences between household and domestic exposures often difficult to discern. Efforts to measure these types of exposure would benefit from clearer definitions; for example, consider defining household exposure as encounters with asbestos-contained structures and materials in built environment, while defining domestic exposure as exposure to asbestos through social interaction with other occupationally exposed human beings. Besides the challenge of having a consistent definition and terminology for various exposure sources, it can be difficult to classify exposure pathway pertinent to a particular study because exposure varies as a function of the lifestyle, social, and environmental factors that bring individuals into contact with asbestos. Individuals may be exposed to asbestos by multiple non-occupational sources, so that it may be difficult or even impossible to separate into distinct exposure pathways.

We identified two common sources from which controls were selected: population-based and hospital-based records. In general, population-based controls are preferred because they offer a better counterfactual for estimating the relative prevalence of asbestos exposure among individuals who did not develop mesothelioma. On the other hand, using hospital- or registry-based controls has the advantage of minimizing the heterogeneity between cases and controls. Among the six case-control studies that we identified as Tier 1, five used population-based controls and one used registry-based controls.

Our meta-analysis of multiple studies suggested that all types of non-occupational asbestos exposure are associated with increased risk of mesothelioma, with varying magnitude of associations and high between-study heterogeneity that is significant. The sources of heterogeneity cannot be fully explained through subgroup analyses, but note that limited studies are included in some of the subgroups. In the subgroup analysis by gender, we found that risk of mesothelioma varied by gender for different types of asbestos exposure, but females had a higher combined RR for any non-occupational exposures and for all non-occupational exposure types than males, and the RR for females was statistically significant different from 1. Subgroup analysis by fiber groups was performed but should be interpreted with caution because only a very limited number of studies reported specific fiber types involved and many exposures were involved multiple fiber types. Misclassification of the fiber type is also possible, for example, studies conducted in earlier years may have misclassified erionite as tremolite. Among the three studies that examined chrysotile-specific risk, a summarized RR estimate for any non-occupational exposure was 3.56 with 95%CI (0.65, 20.82), which is not statistically significantly different from 1. Risk estimates from studies with mixed fiber types showed a much larger RR estimates compared to other groups. In the subgroup analysis by study sites showed varying magnitudes of the association; two studies involved South Africa and Egypt reported a consistent large RR compares to RR studies from other geographic locations. Given between-study heterogeneity remains to be high for most of the subgroup analyses and some summarized RRs had wide confidence interval, these results should be interpreted with caution.

Similar to other review articles, [6–9] results from our study also demonstrated a positive and significant association between the development of mesothelioma and non-occupational asbestos exposure. In comparison, our study examined the relationship among the risk of mesothelioma (not specifically distinguish pleural and peritoneal types) and different pathways of non-occupational asbestos exposures (neighborhood, domestic and household) separately. Because the source and impact of these different exposure pathways can be considerably different from each other, we believe it is important to consider the risk of mesothelioma from each of these exposure routes if possible to provide more insight. Moreover, we included all the studies over several decades that met our selection criterion without restricting to only publications from recent years; we performed a meta-analysis to synthesize quantitatively the risk estimates across multiple studies, including subgroups analyses such as by study quality, using a random-effects model that provides more statistical validity when between-study variability is expected to be large. In addition to meta-analysis, we have also reviewed methods used for exposure assessment and recruitment of controls for case control studies.

It is important to recognize other potential limitations of the current investigation when interpreting the study findings. The studies available for the meta-analysis were few in number. Some of the selected studies had small sample sizes, resulting in large uncertainty in the individual risk estimate. Large heterogeneity between studies, with I^2^ statistic above 80% for all types of exposure, may stem from unclear and often overlapping definitions for non-occupational exposures used by the researchers, which makes interpreting and synthesizing results across studies difficult. Moreover, many studies did not report information on key study characteristics such as pleural versus peritoneal mesothelioma that further limited our ability to perform adequate subgroup analysis based on those characteristics. With the high heterogeneity across the studies, the summary estimate should be interpreted with cautions. The funnel plots suggested a lack of studies with negative associations; thus the results may be at risk of publication bias. However, it is also plausible that it is impossible to have studies with negative association because there is a strong true and positive association. The strong and well-established association between the occupational asbestos exposure and mesothelioma risk supports the second alternative.

## Conclusions

Through this systematic review and meta-analysis of 27 case-control and cohort studies, we have summarized commonly used methods to assess asbestos exposures, and selection of controls. We have also found a positive and strong association between non-occupational asbestos exposure and the risk of mesothelioma. The summarized relative risk estimates vary by types of exposure (neighborhood exposure, domestic exposure, and household exposure) but for all there is an elevated risk of mesothelioma. We observed a large heterogeneity among the selected studies and cannot completely rule out the potential of publication bias. Therefore, caution is needed in interpreting the reported findings. Clear and universally accepted definitions for the different types of non-occupational exposures to asbestos and rigorously conducted studies are warranted to further our understanding of the relationships between non-occupational asbestos exposure and mesothelioma.

## Abbreviation

CC: case-control
CI: confidence interval
MOOSE: Meta-analysis Of Observational Studies in Epidemiology
NOA: naturally occurring asbestos
OR: odds ratio
PRISMA: Preferred Reporting Items for Systematic Reviews and Meta-Analyses (PRISMA)
RR: relative risk
SIR: standardized incidence ratio
SRRE: summary relative risk estimate
